# Mycotoxin Contamination of Edible Non-Timber Forest Products in Cameroon

**DOI:** 10.3390/toxins11070430

**Published:** 2019-07-22

**Authors:** Joseph Fovo Djeugap, Sita Ghimire, Immaculate Wanjuki, Anne Muiruri, Jagger Harvey

**Affiliations:** 1 Phytopathology and Agricultural Zoology Research Unit, Plant Protection Department, Faculty of Agronomy and Agricultural Sciences, University of Dschang, Dschang, Republic of Cameroon; 2 Biosciences eastern and central Africa-International Livestock Research Institute (BecA-ILRI) Hub, Nairobi 30709, Kenya; 3 Texas Feed and Fertilizer Control Service, Office of the Texas State Chemist, Texas A&M, AgriLife Research, Texas A & M University, College Station, TX 77843, USA; 4 Feed the Future Innovation Lab for the Reduction of Post-Harvest Loss, and Department of Plant Pathology, Kansas State University, Manhattan, KS 66506, USA

**Keywords:** Aflatoxin, Edible non-timber forest products, Enzyme-linked immunosorbent assay (ELISA), Fumonisin, VICAM AflaTest, Zearalenone

## Abstract

The prevalence and concentrations of three major mycotoxins, total aflatoxin (AFs), fumonisin (F), and zearalenone (ZEN), were determined on seven edible non-timber forest products (ENTFP) in Cameroon. A total of 210 samples consiting of 30 samples from each ENTFP commodity was collected from farmers and local markets in three agroecological zones of Cameroon and analyzed for moisture content and mycotoxins. Mycotoxins were analyzed using commercial enzyme-linked immunosorbent assay (ELISA) kits and results were validated using the VICAM fluorometric method. The European Union regulation of mycotoxins for human consumption (N°1881/2006) was adopted as reference. The moisture content of samples varied from 5.0% to 22.6%. Aflatoxin contamination was detected in 84.3% samples and only 5.7% exceeded the legal limit (10 ppb). Similarly, 53% of samples were contaminated with fumonisin and 5% of samples exceeded the legal limit (1000 ppb). Zearalenone contamination was detected in 92% of samples and 21% of samples exceeded the legal limit (100 ppb). This is the first report on mycotoxin contamination of ENTFP in the Congo Basin forest. The findings of this study will form a basis for educating farmers and other stakeholders of ENTFP values chain on mycotoxins and mycotoxin mitigation measures to produce safe ENTFP for local and international markets.

## 1. Introduction

In recent years, interest in the potential role of non-timber forest products in reduction of poverty, improvement of nutrition and health, and sustainable management of forest resources has increased [1,2]. The Congo Basin forest, the second largest forest in the world, offers a diversity of edible non-timber forest products (ENTFP). These products serve as a source of food and medicine for more than 65 million people living in or near the forest. The ENTFP in Cameroon’s forests are diverse and exist in many forms: buds, leaves, stems, bark, fruits, seeds, nuts, bulbs, rhizomes, palm wine, tubers, and edible mushrooms [3,4]. In each town nationwide, they are used as alternative sources of food for humans during food shortage [5]. They also reach the European Union markets, generally in forms of vegetables or spices. For example, in France and Belgium, the annual trade of *Gnetum* spp. leaves and *Irvingia* spp. kernels is estimated at US$ 12 and 8 million, respectively [6]. If ENTFPs are well studied and sustainably managed, their production could increase the average income of people living in rural areas and subsequently increase the national average economy. Thus, they have the potential to alleviate poverty among those living in forest zones. 

Many molds belonging to the genus *Aspergillus, Fusarium,* and *Penicillium* are able to colonize ENTFPs, such as grains of *Garcinia kola*, *Monodora myristica,* and *Ricinodendon heudelotii* during storage [7,8]. If the infecting fungi are capable of producing mycotoxins, this could potentially leave consumers vulnerable to risk of mycotoxin exposure. Past studies on ENTFPs, in Cameroon, were focused on their ethnobotanical and medicinal properties [9,10], domestication [11,12], legal and institutional framework [13], and trade [14]. Previous research established the presence of harmful mycotoxins on crops like peanuts and maize [15,16], poultry feed [17], dairy feed, and milk [18], and on tree borne seeds [19]. Given the economic importance of postharvest diseases, along with damage and losses, research on mycotoxin contamination of ENTFPs in Central Africa is critically needed.

According to the International Agency for Research on Cancer [20], Aflatoxin B_1_ and fumonisin are potent carcinogens and toxic to humans and livestock. There is also a clear evidence of genotoxicity due to zearalenone in food especially on laboratory animals and livestock such as pigs and poultry [21]. Ecological factors that are key determinants of aflatoxin accumulation in maize, peanuts, and others crops are temperature and moisture content [22,23]. The objective of this study was to detect and quantify mycotoxins such as aflatoxin, fumonisin, and zearalenone on seven ENTFP species; results can then inform an integrated control strategy to reduce post-harvest losses, protect consumer health, promote export, and increase average income for people living in poverty near or in the forest zones.

## 2. Results

### 2.1. Moisture Content

The mean moisture content was significantly higher (*P* < 0.05) in *Tetrapleura tetraptera* (20.84%) compared to all other commodities (5.29% to 12.09%) with *Ricinodendron heudelotii* having the lowest levels (5.29%) (Table 1). In the Bimodal Rain Forest Zone, moisture content of samples ranged from 5.24% (*R. heudelotii*) to 21.36% (*T. tetraptera*), while in the Monomodal Rainforest Zone it ranged from 5.29% (*R. heudelotii*) to 22.55% (*T. tetraptera*). In the Western High Plateau, the moisture content of samples ranged from 5.11% (*Irvingia gabonensis*) to 18.62% (*T. tetraptera*). 

### 2.2. Total Aflatoxin Contamination of Edible Non-Timber Forest Products

In reference to commodities, 5.56% of samples of *A. lepidophyllus*, 8.33% of samples of *X. aethiopica* and 11.11% of samples of *I. gabonensis* had contamination above the regulatory limit (>10 pbb). *Ricinodendron heudelotii*, *M. myristica,* and *T. tetraptera* had total aflatoxin levels below the regulatory limit (Table 2). The mean aflatoxin content was significantly higher (P < 0.05) in kernels of *I. gabonensis* (3.54 ppb) compared to the others and lowest in *A. melegueta* (0.32 ppb). When considering Agroecological Zones (AEZs), all the commodities and AEZs were positive for Aflatoxin. 

**Figure 1:**
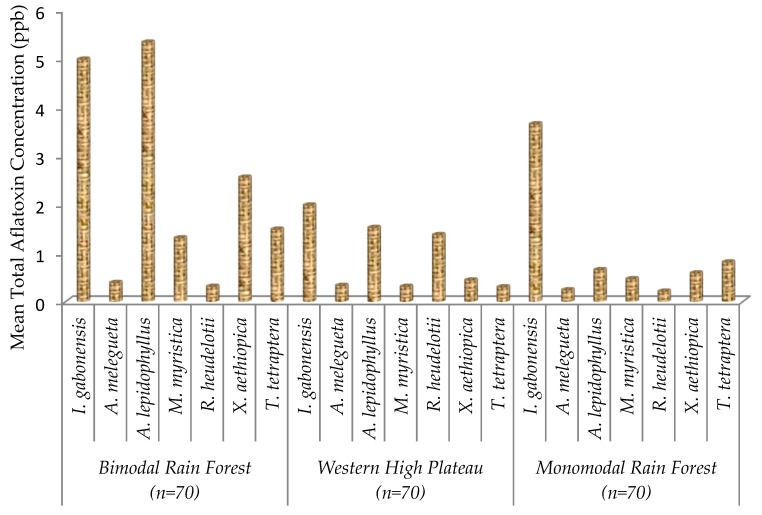
Total aflatoxin content of seven edible non-timber forest products from three agroecological zones in Cameroon.

**Figure 2:**
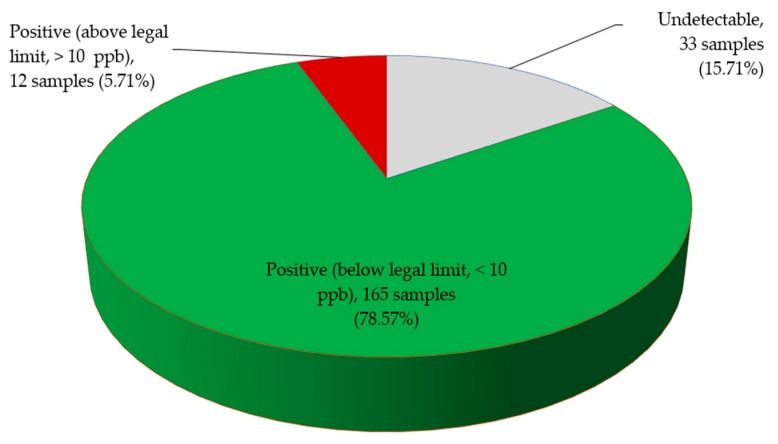
Total aflatoxin levels in edible non-timber forest products (n = 210) collected from farmers and local markets in Cameroon.

Total aflatoxin content in *I. gabonensis* and *A. lepidophyllus* samples from the Bimodal Rainforest (4.98 and 5.33 ppb) was higher than in those collected from the Monomodal Rainforest (3.65 and 0.65 ppb) and Western High Plateau (1.99 and 0.65 ppb), respectively. Occurrence of aflatoxin in *A. melegueta* and *M. myristica* was lower in all the three AEZs (Figure 1). Over 84% of all the samples had detectable levels of aflatoxin, among which only 5.71% were above the regulatory limit. The majority of commodity samples (94.28%) were safe for human consumption (Figure 2). 

### 2.3. Fumonisin Contamination of Edible Non-Timber Forest Products

Fumonisin was not detected in *I. gabonensis* and *M. myristica* samples. The level of fumonisin was significantly higher in samples of *X. aethiopica* (891.97 ppb) follow by *T. tetraptera* (437.08 ppb). All samples of *X. aethiopica* had Fumonisin content above 100 ppb (Table 3).

**Figure 3:**
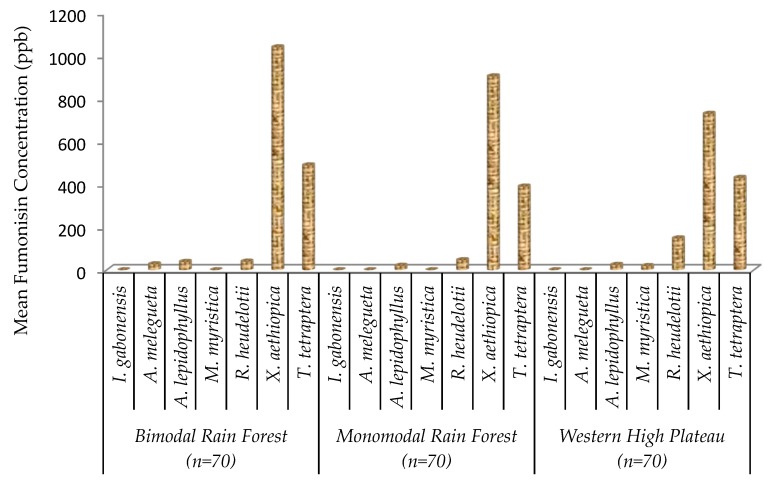
Fumonisin content of seven edible non-timber forest products from three agroecological zones in Cameroon.

**Figure 4:**
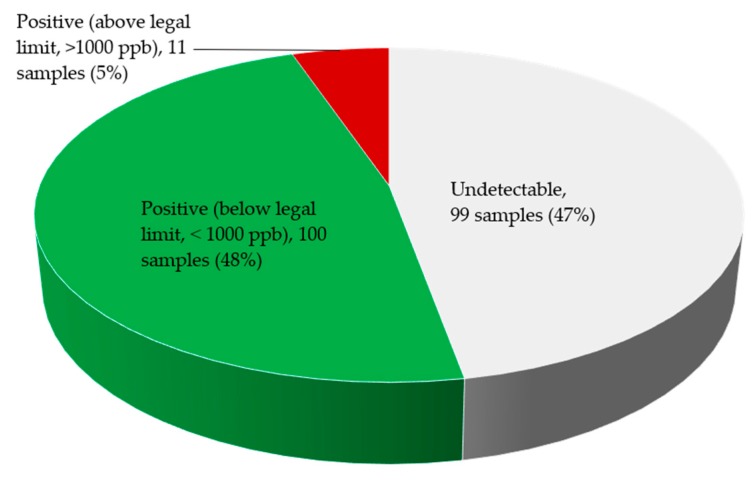
Fumonisin levels in edible non timber forest products (n = 210) collected from farmers and local markets in Cameroon.

Fumonisin content was higher in *X. aethiopica* and *T. tetraptera* samples irrespective of AEZs (Figure 3). Among 210 samples collected from three agroecological zones, 53% had detectable levels of fumonisin and only 5% were above the regulatory limit (>1000 ppb) while 47% were below the limit of detection for fumonisin (Figure 4). 

### 2.4. Zearalenone Contamination on Edible Non-Timber Forest Products

Despite the fact that the regulatory limit of this mycotoxin in food is high relative to some other mycotoxins (100 ppb), the level of zearalenone in some commodities was above the regulatory limit. This was the case for *A. lepidophyllus* (5.55%), *M. myristica* (66.67%), and *X. aethiopica* (100%). The level of zearalenone was significantly higher (P < 0.05) in grains of *X. aethiopica* (219.47 ppb) followed by grains of *M. myristica* (110.89 ppb) than in the other commodities (Table 4). 

**Figure 5:**
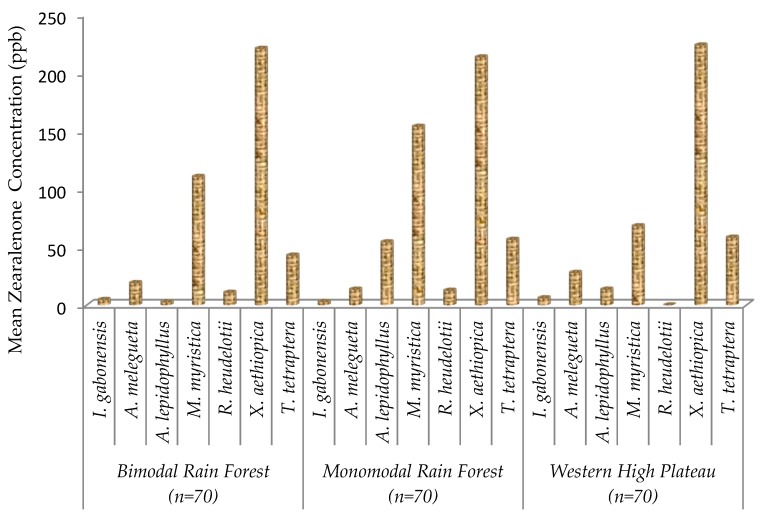
Zearalenone content of seven edible non-timber forest products from three agroecological zones in Cameroon.

**Figure 6:**
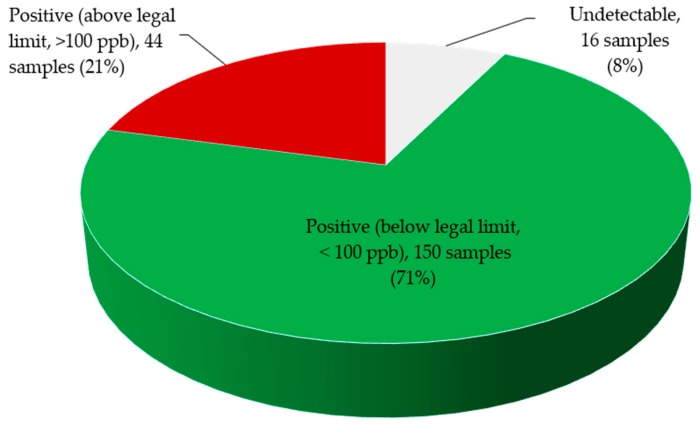
Zearalenone levels in edible non timber forest products (n = 210) collected from farmers and local markets in Cameroon.

Zearalenone content was higher in *X. aethiopica* and *M. myristica* samples irrespective of AEZs; however, the level of this mycotoxin in each commodity did not vary significantly between AEZs (Figure 5). Overall, 92% of samples were positive to zearalenone among which 21% were above the legal limit (>100 ppb), and this mycotoxin was not detected in 8% of the samples (Figure 6).

## 3. Discussion

Mycotoxins are toxic low molecular weight compounds produced by fungi that often contaminate food and feed. Regulations minimizing human exposure to mycotoxins result in high cost to producers, processors, and traders of foodstuffs. Mycotoxin production especially on grains is highly dependent on environmental factors, pre- and postharvest handling, as well as storage conditions.

In this study, the mean moisture content of ENTFP was high (12.67% to 22.55%) in some commodities, which consequently favors growth of mycotoxigenic fungi and consequent contamination with mycotoxins. The higher moisture content could be due to either the chemical composition of the product or the climatic condition in a given agro-ecological zone (AEZ) from where the products were collected, as well as drying practices. In general, the level of total aflatoxin was high in the Bimodal Rain Forest zone for all the commodities except in *R. heudelotii* while the level of fumonisin and zearalenone did not vary between the Monomodal Rain Forest and Western High Plateau zones for all the commodities. In fact, in Cameroon, the Rainforest AEZ is characterized by rainfall between 1,200 and 2,000 mm per year from March to November, temperature ranges from 22 to 32 °C and relative humidity around 80%. Also, the Western High Plateau AEZ is characterized by rainfall from 1,000 to 2,000 mm per annum, with temperatures ranging from 18 to 25 °C and the relative humidity generally over 80%. These climatic conditions favor moisture penetration in many foodstuffs during storage and therefore favors contamination by mycotoxigenic fungi [23,24,25]. Moisture content of the grains is one of the key factors that determine mycotoxin contamination [23], and moisture reduction through proper drying is one of the key mitigation measures. 

Storage fungi usually grow in grains with relative humidity ranging from 70% to 90%, which corresponds to less than 18% moisture content. The most agronomically important fungal genera are *Aspergillus* and *Penicillium*; they are frequently associated with crops in the field and during post-harvest [26]. All the seven edible non-timber forest products (*I. gabonensis, A. melegueta, A. lepidophyllus, M. myristica, R. heudelotii, X. aethiopica* and *T. tetraptera*) included in this study were found to be naturally contaminated with aflatoxin, fumonisin, and zearalenone. Moreover, most of the samples were stored in poor conditions in the houses of smallholder farmers and in the shops of traders. Poor storage conditions is likely to have contributed to the high proportion of mycotoxin positive samples. 

Although these mycotoxins have been reported in other foodstuffs in Cameroon and other Sub- Saharan African countries [24,27,28] information on mycotoxin contamination of ENTFP is not available. In this study, we documented mycotoxin contamination of ENTFP in the Congo Basin forest, and suggest future studies on factors such as moisture content, relative humidity, temperature, substrate composition, and the occurrence of toxigenic fungi that predispose ENTFP to mycotoxin contaminations. It is also important to assess mycotoxins dynamics across the ENTFP value chain to identify critical point(s) of interventions for effective management of mycotoxin contamination in ENTFP.

Aflatoxin level in some samples of *A. lepidophyllus*, *X. aethiopica* and *I. gabonensis* were high and above the regulatory limit (>10 ppb) while *R. heudelotii*, *M. myristica* and *T. tetraptera* samples had aflatoxin levels below the regulatory limit. Similar results were obtained for peanut meal [17] and *Buchanania lanzan* kernels [29]. High concentrations of aflatoxins were reported in edible and medicinal fruits/seeds of forest origin [30], grapes, and nuts [31], milk and milk products [32]. Aflatoxins are highly carcinogenic causing liver cancer and have also been associated with acute hepatitis in humans, mostly in the developing world [33,34]. 

As far as fumonisin is concerned, all samples of *X. aethiopica* contained fumonisin above the regulatory limit (>100 ppb). The high level of this mycotoxin was also reported by many researchers especially in maize and maize-based foods [35,36] where *Fusarium verticillioides* and *F. proliferatum* were the two main species with the capacity to produce this mycotoxin [37]. Many studies have been carried out on fumonisin because of its cancer promoting activity in humans and the induction of leukoencephalomalacia in horses [38,39]. 

The study also shows that 92% of samples (n = 194) were positive for zearalenone, among which 21% were above the legal limit (>100 ppb), and the zearalenone level was above the regulatory limit in all the samples of *X. aethiopica*. High levels of zearalenone have been reported in corn, wheat, sorghum, barley, oats, sesame seed, and corn silage [40]. In fact, zearalenone is a common mycotoxin of all major cereal grains worldwide specially corn and wheat [41,42]. This toxin is generated in the field or during storage of moist grain contaminated by various *Fusarium* spp. in the pre-harvest period [43]. The accumulation of zearalenone in cereals depends on substrate type, temperature, duration of *Fusarium* growth, and strain of fungal species. Moreover, a humid tropical climate promotes microbial proliferation on food and feedstuffs and thus mycotoxin biosynthesis [44]. Zearalenone has oestrogenic effects in various animal species that include infertility, vulvae oedema, and mammary hypertrophy in females [45].

Given the fact that most mycotoxins are heat stable, cooking of contaminated products does not destroy them. For example, aflatoxins have been found to be heat stable with melting points of between 268 to 269 °C [46]. Hence, the presence of these mycotoxins in ENTFP is a threat to consumers, and the consumption of mycotoxin-contaminated ENTFP should be avoided. 

In this study, a relatively low proportion of the samples had mycotoxin contamination above the regulatory limit for aflatoxin (>10 ppb), fumonisin (>100 ppb), and zearalenone (>100 ppb). It is important to note that appropriate mitigation strategies and technologies are available that can reduce the risk of mycotoxin contamination of these foods, including Good Agricultural Practices, and proper drying and storage. Harvesting products in sunny days, minimizing damage to the products during harvesting, and adoption of appropriate processing methods and storage practices could help to bring the level of the mycotoxin contaminations below the legal limit. Mitigation strategies for other fruits and nuts can readily be adapted for use by smallholder farmers and other value chain actors, to reduce mycotoxin contamination of these commodities in the Congo Basin. This will make these products safe for consumption and promote local, regional, and international trade of ENTFP. Most of the ENTFP are low volume, high value commodity so they can potentially improve the quality of life for people living in the Congo Basin.

## 4. Conclusions

This is the first report on mycotoxin contamination of ENTFP in the Congo Basin forest. Although a high proportion of samples analysed in this study were contaminated with major mycotoxins, only a few samples (5.7% for Aflatoxin, 5% for Fumonisin, and 21% for Zearalenone) had contamination level above the regulatory limits set by the European Union regulation of mycotoxins for human consumption. Moreover, we could not detect aflatoxin, fumonisin, and zearalenone in 15.7%, 47%, and 8% of the samples, respectively suggesting these ENTFP were free or nearly free from these three major mycotoxins. This study shows the importance of introducing mitigation strategies for lowering/preventing mycotoxin contamination in ENTFP by adopting appropriate pre- and post-harvest practices. Therefore, creating the awareness of farmers, traders, consumers, and other relevant stakeholders of the ENTFP value chain on mycotoxin contamination, associated health risks, and mitigation measures (e.g. harvesting crops at maturity, minimizing damage during harvest, proper drying, appropriate packaging and storage to prevent moisture) should be a high priority. Studies documenting traditional practices of harvesting and post-harvest storage and processing practices of ENTFPs are important to identify technology gaps for producing healthy ENTFP products. Also, it is important to identify fungal species responsible for mycotoxin production in the ENTFP in Cameroon, and to develop integrated management strategies to minimize fungal colonization and mycotoxin contamination of the socioeconomic important edible non-timber forest products. Such collective efforts will underpin the production of safe ENTFPs for local consumption, as well as promote trade of ENTFP in local, regional, and international markets.

## 5. Materials and Methods

### 5.1. Sample Collection Areas 

Five agroecological zones (AEZs) are present in Cameroon that include: Sudano-Sahelian (zone I) in the North and Extreme North region, Sudano-Guinea (zone II) in the Adamaoua Plateau, Western High Plateau (zone III) in the West and North-west region, Humid Forest with unimodal rainfalls (zone IV) in the Littoral and South-west region, and the Humid Forest with bimodal rainfalls (zone V) in the Central and Eastern part of the country. In this study, samples were collected in three AEZs (zones III, IV and V) chosen based on ENTFP significance in terms of production in the country. The Western High Plateau (III) lies at altitudes ranging between 1100 m and 2000 m above sea level (ASL). The rainfall distribution is bimodal and ranges from 1000 to 2000 mm per annum, with two cropping seasons in the valley and one in the mountain areas. This is the coolest part of the country, with temperatures ranging from 18 to 25 °C. The long wet season of 8–9 months spans from March to November and the high humidity generally over 80% ensures continuous presence of moisture. The Rainforest AEZ (IV and V) in the southern part of the country lies at altitudes ranging between 0 and 800 m ASL. This AEZ is characterized by forest/Savanna mosaic vegetation with rainfall between 1200 and 2000 mm per year from March to November. The temperature ranges from 22 to 32°C and the relative humidity is normally around 80%.

### 5.2. Sampling 

A total of 210 samples of ENTFP (kernels of Irvingia gabonensis, grains of Aframomum melegueta, Afrostyrax lepidophyllus, Monodora myristica, Ricinodendron heudelotii, and dry fruit of Xylopia aethiopica and Tetrapleura tetraptera) were randomly collected from smallholder farmers and local markets in six localities (2 per AEZ) and kept in plastic sacks, sealed under vacuum and stored at 4 °C until analysis. Localities chosen were separated by a minimum distance of 100 km. For each commodity, five samples (150 g each) were collected per locality that gave a total of 30 samples for each product.

### 5.3. Determination of Moisture Content

**Figure 7:**
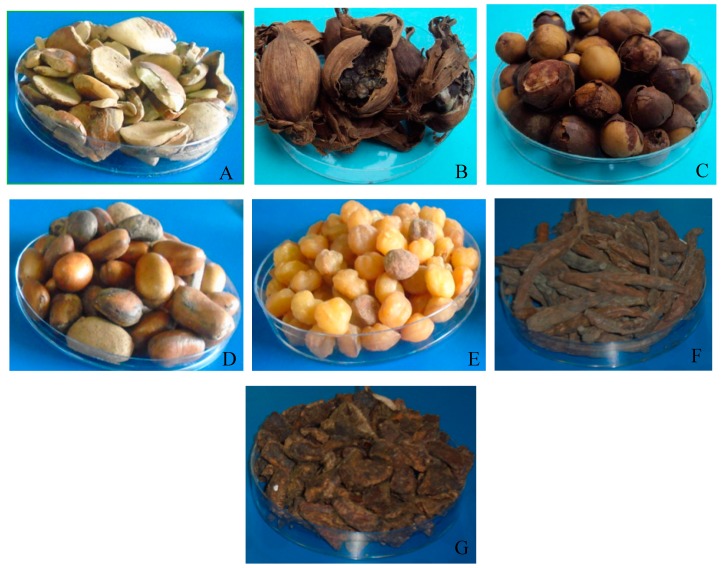
Edible non-timber forest products (spices, grains and fruits) tested for mycotoxins: *Irvingia gabonensis* (**A**), *Aframomum melegueta* (**B**), *Afrostyrax lepidophyllus* (**C**), Monodora myristica (**D**), *Ricinodendron heudelotii* (**E**), *Xylopia aethiopica* (**F**) and *Tetrapleura tetraptera* (**G**).

Moisture content of plant product samples (Figure 7) was determined using the standard oven drying method [46]. Samples were weighed, dried in triplicate at 100 °C for 6 hours to constant weight in an oven (Gallenkamp Oven BS), and the mean moisture content was calculated on a percentage dry weight basis.

### 5.4. Mycotoxin Extraction Procedures

Dried grains or kernels of each commodity were ground separately in the laboratory in a Romer Mill (Romer Labs ®, Inc., Union, MO) which was disinfected with 10% bleach after each sample to avoid cross contamination and stored in a cool room. 

#### 5.4.1. Total Aflatoxin Extraction

The common Total Aflatoxin extraction protocol used for corn, wheat, hay, snaplage, paprika, pistachio, and peanut was used for ENTFP, based on their matrix being similar to pistachio and peanut. In brief, 150 g of each sample was ground into fine powder, mixed, and 5.0 g of each was introduced into a 50 mL falcon tube. Then, 25 mL of 80% methanol was added to the tubes with the samples at a ratio of 1:5 (w/v), mixed and shaken vigorously at 150 revolutions per min for 2 min using a laboratory shaker. Extracts were filtered through a Whatman N°1 filter paper. Then, an aliquot of the filtrate was diluted in a ratio of 1:10 with reconstituted wash buffer (PBS). 

#### 5.4.2. Fumonisin Extraction

For fumonisin extraction, 5.0 g of flour of each ENTFP was introduced in a 50 mL falcon tube. Then, 10 mL of extraction solvent (90% methanol) was added into the tube at a ratio of 1:2 (w/v), mixed, shaked, and filtered as described previously. Then, an aliquot of the filtrate was diluted with distilled water at a ratio of 1:20.

#### 5.4.3. Zearalenone Extraction

Extraction of zearalenone was carried out by adding 25 mL of 70% methanol in the falcon tubes containing 5.0 g of samples at a ratio of 1:5 (w/v). After mixing, shaking and filtering, an aliquot of the filtrate was diluted with 70% methanol at a ratio of 1:10.

### 5.5. Mycotoxin Quantification 

Each mycotoxin was analyzed using a commercial enzyme-linked immunosorbent assay (ELISA) kits following the manufacturer’s protocol (Helica Biosystems Inc., Fullerton, CA, USA). Total Aflatoxin Assay-low matrix ELISA kit (Helica catalog number 981AFL01LM-96), Fumonisin ELISA kit (Helica catalog number 951FUM01C-96) and Zearalenone ELISA kit (Helica catalog number 951ZEA01N-96), with 96-well antibody-coated microplates coated, were used to analyze and quantify each type of mycotoxin. The optical density of each antibody coated microtiter well was read at 450 nm using a microplate reader (BioTek Instruments, Inc., Winooski, VT, USA). Test values were interpreted with reference to standards that were included in each experiment. The lower and upper limits of quantification of total aflatoxin, fumonisin, and zearalenone with the ELISA kit were 1 and 20 ppb, 100 and 6000 ppb and 15 and 500 ppb, respectively. Samples with AFs, F, or ZEN levels below the quantification limit were considered as containing no detectable mycotoxin while samples with AFs, F, or ZEN levels above the quantification limit were diluted and re-tested. 

### 5.6. Validation of ELISA Data

Data from ELISA techniques were validated by re-analysing a randomly selected subset of 40 samples using the VICAM AflaTest (Watertown, MA, USA), a flourometric-immunocapture assay. Validation was done only for aflatoxin. The extraction procedure for VICAM analysis was as follows: 5.0 g of powder of each sample was weighed, mixed with 0.5 g sodium chloride, added with 10 mL of 80% methanol solution (methanol:water, 80:20 v/v) and incubated at 25 °C for 4 min at 225 rpm in a controlled environment shaker (New Brunswick Co. Inc, Edison, NJ, USA). The mixture was filtered using fluted filter paper (Folder Grade 1289, VICAM, A Waters Business), 2 mL of filtrate was diluted with 8 mL of distilled water in a clean tube, and mixed for 2 min on a Denley Spiramix Linear Mixer (Denley, Sussex, UK). Two mL of diluted filtrate (0.2 g sample equivalent) was passed through an Aflatest®-P affinity column at a rate of 1 to 2 drops/second and the column was then rinsed twice with 5 mL of distilled water at the same rate. The aflatoxin material bound to the affinity column was eluted with 1 mL of absolute methanol at the rate of 1 to 2 drops/second and the eluate was collected in a glass tube. For quantification of aflatoxin, 1 mL of Aflatest^®^ developer solution (made using 1 mL developer: 9 mL distilled water) was added to the eluate in a glass tube from the Aflatest-P column. The glass tube was agitated to mix and concentration of total aflatoxin (ppb) was read after 60 seconds using a VICAM fluorometer (Series-4EX, Source Scientific LLC, USA) calibrated with a methanol blank following the manufacturer’s protocol. 

### 5.7. Data Analysis

Data were subjected to one-way analysis of variance (ANOVA) to determine the significance of the results and regression/correlation analysis was used to determine R^2^. Statistical analyses were carried out using SAS software (version 9.1) and the Duncan multiple range test was used to determine differences in the means among samples obtained from the different AEZs at P = 0.05. The European Union regulation (N°1881/2006) of mycotoxins for human consumption was used as reference for data analysis.

## Figures and Tables

**Table 1:** Moisture content of different edible non-timber forest products species from three agroecological zones of Cameroon.

Commodities	Agroecological Zones (AEZs)	Number of Samples	Mean within AEZs (%)	Mean (%)
*Irvingia gabonensis* (n = 30)	Bimodal Rainforest	10	5.94	5.83 ± 0.6 ^c^
	Monomodal Rainforest	10	6.43	
	Western High Plateau	10	5.11	
*Ricinodendron heudelotii* (n = 30)	Bimodal Rainforest	10	5.24	5.29 ± 0.04 ^c^
	Monomodal Rainforest	10	5.29	
	Western High Plateau	10	5.33	
*Afrostyrax lepidophyllus* (n = 30)	Bimodal Rainforest	10	10.66	12.03 ± 1.1 ^b^
	Monomodal Rainforest	10	12.67	
	Western High Plateau	10	12.77	
*Aframomum melegueta* (n = 30)	Bimodal Rainforest	10	13.52	12.09 ± 1.6 ^b^
	Monomodal Rainforest	10	11.71	
	Western High Plateau	10	11.06	
*Monodora myristica* (n = 30)	Bimodal Rainforest	10	9.46	10.08 ± 1.5 ^b^
	Monomodal Rainforest	10	11.62	
	Western High Plateau	10	9.15	
*Xylopia aethiopica* (n = 30)	Bimodal Rainforest	10	10.48	6.99 ± 2.2 ^c^
	Monomodal Rainforest	10	5.47	
	Western High Plateau	10	5.01	
*Tetrapleura tetraptera* (n = 30)	Bimodal Rainforest	10	21.36	20.84 ± 1.6 ^a^
	Monomodal Rainforest	10	22.55	
	Western High Plateau	10	18.62	

^a,b,c^ Means within a column with different superscripts are significantly different at P < 0.05.

**Table 2:** Concentration of total aflatoxin (ppb) in the edible non timber forest products from Cameroon.

Commodities	Percentage of Positive Samples		Mean (ppb)
*Irvingia gabonensis*	<1 ppb	11.11%	3.54 ± 0.9 ^a^
	1–10 ppb	77.77%	
	10–20 ppb	11.11%	
*Aframomum melegueta*	<1 ppb	94.44%	0.32 ± 0.1 ^c^
	1–10 ppb	5.56%	
	10–20 ppb	0%	
*Afrostyrax lepidophyllus*	<1 ppb	55.55%	2.5 ± 0.7 ^a,b^
	1–10 ppb	38.9%	
	10–20 ppb	5.56%	
*Monodora myristica*	<1 ppb	94.44%	0.7 ± 0.2 ^c,b^
	1–10 ppb	5.56%	
	10–20 ppb	0%	
*Ricinodendron heudelotii*	<1 ppb	83.33%	0.63 ± 0.2 ^c,b^
	1–10 ppb	16.67%	
	10–20 ppb	0%	
*Xylopia aethiopica*	<1 ppb	58.33%	1.2 ± 0.2 ^c,b^
	1–10 ppb	33.33%	
	10–20 ppb	8.33%	
*Tetrapleura tetraptera*	<1 ppb	72.22%	0.9 ± 0.4 ^c,b^
	1–10 ppb	27.78%	
	10–20 ppb	0%	

^a,b,c^ Means within a column with different superscripts are significantly different at P < 0.05.

**Table 3:** Concentration of fumonisin (ppb) in the edible non-timber forest products from Cameroon.

Commodities	Percentage of Positive Samples		Mean (ppb)
*Irvingia gabonensis*	<100 ppb	0%	0.00 ^f^
	100–1000 ppb	0%	
	1000–6000 ppb	0%	
*Aframomum melegueta*	<100 ppb	94.44%	9.30 ± 2.4 ^e^
	100–1000 ppb	5.56%	
	1000–6000 ppb	0%	
*Afrostyrax lepidophyllus*	<100 ppb	94.44%	28.23 ± 8.7 ^d^
	100–1000 ppb	5.56%	
	1000–6000 ppb	0%	
*Monodora myristica*	<100 ppb	100%	6.52 ± 2.9 ^e^
	100–1000 ppb	0%	
	1000–6000 ppb	0%	
*Ricinodendron heudelotii*	<100 ppb	94.44%	78.62 ± 12.3 ^c^
	100–1000 ppb	5.56%	
	1000–6000 ppb	0%	
*Xylopia aethiopica*	<100 ppb	0%	891.97 ± 84.9 ^a^
	100–1000 ppb	50.0%	
	1000–6000 ppb	50.0%	
*Tetrapleura tetraptera*	<100 ppb	11.11%	437.08 ± 78.6 ^b^
	100–1000 ppb	83.33%	
	1000–6000 ppb	5.55%	

^a,b,c^ Means within a column with different superscripts are significantly different at P < 0.05.

**Table 4:** Concentration of zearalenone (ppb) in the edible non-timber forest products from Cameroon.

Commodities	Percentage of Positive Samples		Mean (ppb)
*Irvingia gabonensis*	<15 ppb	100%	4.61 ± 1.6 ^d^
	15–100 ppb	0%	
	100–500 ppb	0%	
*Aframomum melegueta*	<15 ppb	44.44%	20.24 ± 7.7 ^c,d^
	15–100 ppb	55.56%	
	100–500 ppb	0%	
*Afrostyrax lepidophyllus*	<15 ppb	77.78%	23.51 ± 9.8 ^c,d^
	15–100 ppb	16.67%	
	100–500 ppb	5.55%	
*Monodora myristica*	<15 ppb	0%	110.89 ± 22.7 ^b^
	15–100 ppb	33.33%	
	100–500 ppb	66.67%	
*Ricinodendron heudelotii*	<15 ppb	77.78%	7.84 ± 3.5 ^d^
	15–100 ppb	22.22%	
	100–500 ppb	0%	
*Xylopia aethiopica*	<15 ppb	0%	219.47 ± 35.2 ^a^
	15–100 ppb	0%	
	100–500 ppb	100%	
*Tetrapleura tetraptera*	<15 ppb	0%	52.56 ± 16.1 ^c^
	15–100 ppb	100%	
	100–500 ppb	0%	

^a,b,c^ Means within a column with different superscripts are significantly different at P < 0.05.
